# Neonatologists' Perspectives on Exploring Parental Spirituality in Prenatal Consultations

**DOI:** 10.1089/pmr.2022.0052

**Published:** 2023-03-30

**Authors:** Dana L. Peralta, Dominic Moore, Ryann Bierer

**Affiliations:** ^1^ Department of Pediatrics, University of Utah School of Medicine, Salt Lake City, Utah, USA.; ^2^ Division of Pediatric Palliative Care, Department of Pediatrics, University of Utah School of Medicine, Salt Lake City, Utah, USA.; ^3^ Division of Neonatology, Department of Pediatrics, University of Utah School of Medicine, Salt Lake City, Utah, USA.

**Keywords:** decision making, prenatal, religion, spirituality, values

## Abstract

**Background and Objectives:**

Values of religion, spirituality, and faith (RSF) are central to decision making for many parents facing extremely preterm labor or prenatal diagnoses of potentially life-limiting congenital anomalies. Neonatologists' opinions and comfort with discussing parental RSF are not well known. We sought to understand neonatologists' current practices and perceptions of exploring parental RSF in prenatal consultations.

**Methods:**

A retrospective chart review was performed at a single U.S. academic institution to evaluate the inclusion of spiritual terminology in documentation. All mothers who were admitted with anticipated extremely preterm delivery as well as those with prenatal diagnoses of potentially life-limiting congenital anomalies were included in analysis. After chart review, an anonymous survey was distributed to neonatology attendings and fellows to examine perspectives on exploring parental RSF.

**Results:**

The chart review indicated that RSF terminology was absent from the documentation of all prenatal consultations performed by neonatology. Sixty-five percent of survey respondents considered RSF important in their personal lives and 47% considered RSF important in clinical practice. The three most significant barriers to exploring RSF were lack of training or education in spiritual care, differences between physicians' and patients' personal beliefs, and insufficient time.

**Conclusions:**

Our study highlights a gap between the goal for prenatal counseling in cases of extreme prematurity and potentially life-limiting congenital anomalies and current practices that frequently exclude the values most important to many parents. Lack of training in spiritual care is a significant barrier to neonatologists exploring parental RSF.

## Background

For parents anticipating the birth of a child, few envision preparing for the arrival of that child at the extremes of prematurity or that the child would be diagnosed prenatally with a potentially life-limiting congenital anomaly. Parents in this situation are looking for support in balancing the best medical information alongside their deeply held values, which may include religion and spirituality.

Historically, prenatal counseling has focused on prognostication including delivery-room resuscitation, need for interventions including invasive ventilation, short- and long-term morbidity, anticipated length of stay in the neonatal intensive care unit (NICU), and risk of mortality.^1–5^ Although discussions of survival and potential morbidities are important in prenatal counseling, many studies suggest that these predictions serve a limited role in parents' decision making compared with personal values and preferences.^4,6–9^

Existing literature shows that religion and spirituality serve a multitude of roles for many parents of critically ill children. Spiritual practices of prayer, meditation, and the reading of sacred texts together with faith leaders and communities serve as significant sources of support, comfort, and connectedness through challenging times.^10–15^ In addition, studies have shown that religion and spirituality are foundational to parents' coping, decision making, and finding meaning through the course of their child's illness.^10,11,14,16^ Despite the centrality of these values for many parents, neonatologists place less emphasis on a family's emotional and spiritual needs or values during discussions in the perinatal period.^8,17,18^ This approach results in assessments by neonatologists that lack the full perspective of parents.^19^

The importance of parental values is recognized in current recommendations that state that prenatal counseling should elicit parents' wishes, concerns, as well as values that might guide decisions.^2–5^ Currently, little is known about neonatologists' opinions, practices, and comfort with discussing the parental values of religion and spirituality in the perinatal period.

In this study, we sought to understand neonatologists' perspectives, current practices, and potential barriers to exploring parental religion, spirituality, and faith (RSF) in prenatal consultations of high-risk infants at our institution, a tertiary academic referral center located in the Mountain West region of the United States. We hypothesized that most neonatologists do not routinely discuss parental RSF in prenatal consultations and that there are multiple barriers preventing these discussions.

## Methods

We employed a mixed-methods approach involving two quantitative phases, beginning with a retrospective chart review to determine the frequency of inclusion of RSF in the documentation of prenatal consultations of high-risk infants. Based on the chart review findings that spiritual terminology was absent from neonatologists' documentation, we developed a survey evaluating neonatologists' attitudes and perspectives on exploring parental RSF. The study design was determined to be exempt by the University of Utah Institutional Review Board.

We conducted a retrospective chart review of all mothers who were admitted to the University of Utah Hospital with anticipated extremely preterm delivery (gestational age <25^0/7^ weeks) as well as those with prenatal diagnoses of potentially life-limiting congenital anomalies including anencephaly, trisomy 13 and 18, hypoplastic left heart syndrome, and bilateral renal agenesis during the 5-year period of March 2015 through March 2020.

Eighty-seven mothers with these diagnoses were identified. Mothers were excluded in cases of intrauterine fetal demise as well as cases of extreme prematurity in the absence of congenital anomalies in which gestational age was <22^0/7^ weeks and considered previable, or if delivery occurred at a gestational age >25^0/7^ weeks.

Data were collected on the inclusion of religious and spiritual terminology in prenatal consultations performed by neonatology as well as maternal–fetal medicine, social work, spiritual care, and palliative care documentation. Spiritual terminology included religious affiliation, spiritual rites and practices (prayer, baptism, blessing, and attending religious services), faith leader and faith community support, belief in miracles, maintaining hope and faith, and questioning God or a “Higher Power.” A detailed list of spiritual terminology is available in the Supplementary Material S1. Descriptive statistics were employed to determine the frequency of spiritual and religious terminology in prenatal encounters.

We next sought to understand neonatologists' attitudes, current practices, and barriers to exploring parental RSF. Although there are several validated instruments available to assess physician's religiosity and spirituality,^20–24^ to our knowledge there is no existing tool that examines neonatologist's attitudes toward parents' religion and spirituality in the prenatal consultation. Thus, after review of the literature, we developed a survey assessing neonatologists' perspectives on and barriers to exploring parental RSF in prenatal consultations of high-risk infants.

The survey questions were reviewed by two neonatologists and two pediatric palliative care physicians whose insights were used to improve construct, content, and face validity. The final survey was constructed using Qualtrics™ software and consisted of 14 closed-ended questions divided into three sections. The first section collected respondent demographics. The second section evaluated attitudes and perspectives on RSF in the perinatal period using 5-point Likert scales. The third section sought to identify the most significant barriers to exploring RSF in prenatal consultations of high-risk infants. Open-ended responses were optional for several questions. The survey required ∼5–10 minutes to complete. See Supplementary Materials S1 for survey instrument.

The survey was distributed through email to all neonatology attendings and fellows in the division of neonatology at the University of Utah School of Medicine. Anonymity was preserved using Qualtrics anonymous link that does not collect any identifying information. Data were collected from June 2021 through July 2021. Descriptive analyses were performed, with summary statistics presented as frequencies and proportions.

## Results

### Inclusion of religion, faith, and spirituality in documentation of prenatal consultations

The final chart review included 45 mothers. Twenty-four mothers were anticipating extremely preterm delivery and 21 mothers carried prenatal diagnoses of potentially life-limiting congenital anomalies. RSF content was present in 31% (14/45) of patient documentation. RSF terminology was absent in the documentation of all prenatal consultations performed by neonatology. Social work was involved in all cases and documentation included religious and spiritual content in 22% (10/45) of maternal charts, with religious preference or affiliation representing the majority of this content (8/10, 80%).

**FIG. 1.**
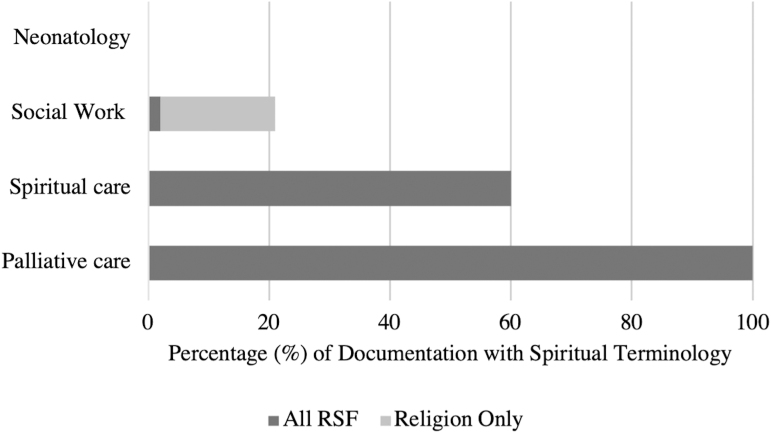
Frequency of RSF content included in documentation by discipline. RSF terminology was absent in documentation of all prenatal consultations by neonatology. Twenty-two percent of all documentation by social work included spirituality terminology, with 80% stating a patient's religious preference or affiliation. All documentation by palliative care included RSF terminology. RSF, religion, spirituality, and faith.

Spiritual care was involved in a minority of cases (10/45, 22%) and palliative care was involved exclusively in cases of congenital anomalies (5/21, 11%). Combined, spiritual care and palliative care were involved in 29% (13/45) of cases. When palliative care was involved, a patient or family's religious background and spiritual beliefs were universally described, even if these values were not important to a family's decision making (Fig. 1).

### Provider survey

#### Demographic data

We analyzed a total of 19 surveys, 17 of which were completed and 2 of which were partially completed. The survey was distributed to 33 neonatology attendings and fellows (58% response). Demographic data for the respondents are summarized in Table 1. The mean age range was 31–40 years (9/19, 47%), and 63% of respondents identified as female. The majority were attendings (16/19, 84%) with 10 or less years in practice (12/19, 64%).

**Table 1.** Demographic Characteristics of Survey Respondents

	***n*** = 19 (%)
Age range, years	
30 or younger	1 (5)
31–40	9 (47)
41–50	4 (21)
51–60	3 (16)
61 and older	2 (11)
Gender	
Male	5 (26)
Female	12 (63)
Nonbinary	0 (0)
Prefer not to say	2 (11)
Role in neonatology	
Attending	16 (84)
Fellow	3 (16)
Years of practice in current role	
5 or less	10 (53)
6–10	2 (11)
11–15	2 (11)
16 or more	5 (26)
Spirituality and religiosity	
Spiritual	8 (42)
Religious	1 (5)
Both	5 (26)
Neither	5 (26)

#### Perspectives on RSF

**FIG. 2.**
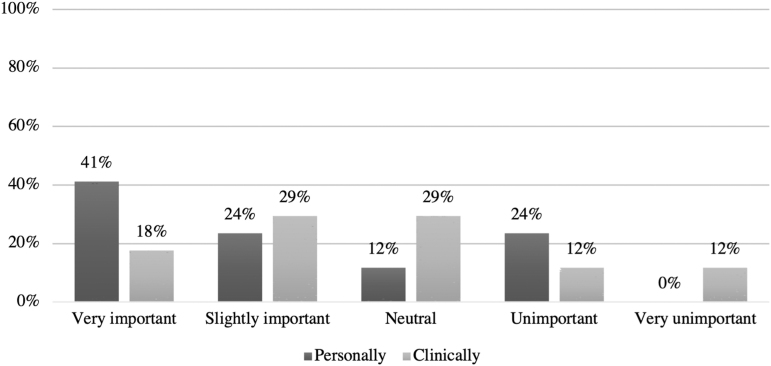
Comparison of the importance of RSF in respondents' personal lives and clinical practice. Sixty-five percent of respondents rated RSF as very or slightly important in their personal lives and 47% considered RSF important in clinical practice. Twenty-four percent of respondents considered RSF unimportant or very unimportant in their personal lives and clinical practice.

Most respondents characterized themselves as spiritual (8/19, 42%) or both spiritual and religious (5/19, 26%). A minority identified as religious (1/19, 5%) or neither spiritual nor religious (5/19, 26%). Only five respondents reported identifying with a specific religious or faith community, including Christian, Protestant, Methodist, and The Church of Jesus Christ of Latter-Day Saints. Sixty-five percent of respondents rated RSF as very or slightly important in their personal lives and 47% considered RSF important in clinical practice. Twenty-four percent of respondents considered RSF unimportant or very unimportant in their personal lives and clinical practice (Fig. 2).

#### Exploring parental RSF

Approximately half (9/17, 53%) of respondents reported that they explore RSF in prenatal consultations for high-risk infants sometimes, often, or always, whereas the other half (8/17, 47%) rarely or never explore RSF in prenatal consultations. At the same time, 70% (12/17) of surveyed neonatologists agreed that it is of some degree of importance for neonatologists to be aware of parents' RSF as it pertains to decision making for high-risk infants. Only 1 in 17 (6%) of respondents asserted that it is unimportant for neonatologists to have this awareness. One respondent posed that it is “only important [for neonatologists to be aware of parental RSF] if the family desires us to know or special accommodations need to be made.”

When considering whose role it is to explore parental RSF in the perinatal period, respondents overwhelmingly agreed that spiritual care (16/17, 94%), social work (15/17, 88%), and palliative care (15/17, 88%) share this responsibility. At the same time, more than half of respondents also believe that it is within the role of neonatology (11/17, 65%), maternal–fetal medicine (10/17, 59%), and the bedside nurse (8/17, 47%). One respondent wrote that it is within the role of “everyone if helpful for the family.”

**FIG. 3.**
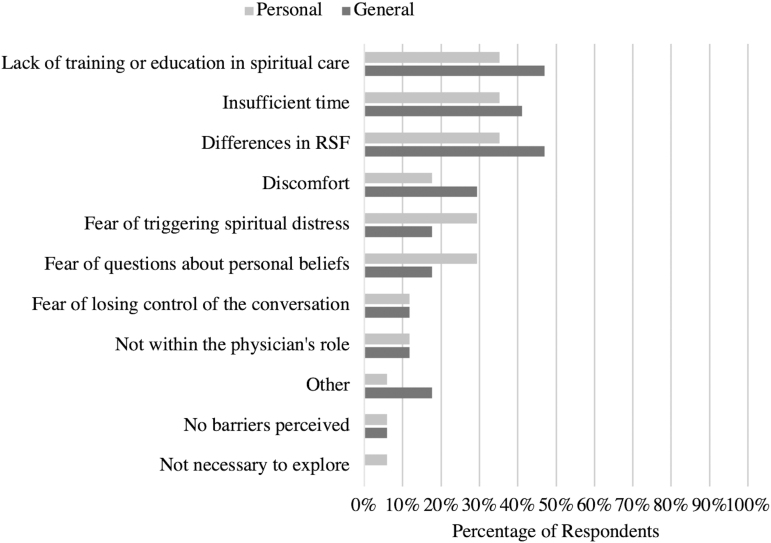
General and personal barriers to exploring parental RSF. Lack of training or education in spiritual care (47% and 35%), differences between physicians' and patients' personal beliefs (47% and 35%), and insufficient time (41% and 35%) were cited as the most significant barriers, generally and personally.

Respondents rated the three most significant barriers, generally and personally, to exploring parental RSF in prenatal consultations (Fig. 3). Lack of training or education in spiritual care (47% and 35%), differences between physicians' and patients' personal beliefs (47% and 35%), and insufficient time (41% and 35%) were cited as the most significant barriers, generally and personally. Discomfort (29% and 18%), fear of triggering spiritual distress in patients (18% and 29%), and fear around answering questions about personal faith beliefs (18% and 29%) were also common responses.

A few respondents elaborated on perceived barriers, stating “Religion/spirituality has potential to either unite or divide any team. I worry sometimes that the family will not appreciate knowing that my beliefs potentially conflict with their own.” Two respondents reported that it was not within the physician's role to explore RSF in prenatal consultations, stating, “I don't think anyone expects me (physician) to explore spiritual matters.” Most respondents (13/17, 76%) expressed interest in learning more about the role of values including RSF in parental decision making, coping, and meaning making.

## Discussion

The prenatal consultation is a life-altering discussion for many parents. Expectant parents face unanticipated difficult decisions regarding delivery-room resuscitation, interventions, and numerous potential complications. Previous studies have shown that religion and spirituality influenced parents' decision making more than physicians' predictions of the infant's morbidity and mortality or the level of detail in which information was presented.^8,18^ In order for neonatologists to provide optimal guidance and support, parents' values, including religion and spirituality, must be uncovered. This study sought to understand neonatologists' current practices, perspectives, and barriers to exploring parental spirituality in the prenatal consultation of high-risk infants.

Our chart review showed that RSF content is rarely included in documentation during the prenatal period. In cases wherein spiritual terminology was included, it was predominantly stated as religious preference or affiliation and gave little insight into the meaning of these values for parents. RSF was absent in the documentation of prenatal consultations by neonatology. Although it is possible that discussions of RSF occurred without being documented, the lack of RSF in neonatologists' documentation suggests that these values are infrequently explored as factors in parents' decision making for high-risk infants.

The absence of RSF in prenatal documentation aligns with the survey's finding that only half of respondents reported exploring parental RSF in prenatal consultations of high-risk infants. This finding supports our hypothesis that exploration of parental RSF is not standard of practice for neonatologists at our institution. Variability in neonatologists' RSF exploration may be explained by neonatologists viewing these discussions as outside of their responsibility.

Previous studies have also shown that physician's attitudes regarding discussion religion and spirituality in the clinical setting are highly variable.^25–27^ Although more than half of respondents agreed that exploration of parental spirituality is within the scope of neonatologists, the majority agreed that these discussions are primarily within the role of spiritual care, social work, and palliative care.

Despite the variability in current practice and perceived responsibility relating to RSF exploration, the majority of neonatologists in our study agree that it is important to have awareness of parents' RSF as it pertains to decision making. If recognition of these parental values is important, then what is preventing neonatologists from exploring RSF? Our study showed that a lack of training or education in spiritual care is a significant barrier. Previous studies have shown that neonatologists are not universally trained nor skilled at exploring parental values, much less in applying these values to the inherent uncertainty of an infant's life.^28–31^

Thus, neonatologists' inconsistency in initiating these discussions as well as transferring the responsibility to other members of the medical team likely reflects neonatologists being unequipped and, therefore, hesitant to delve into the spiritual realm. Additional significant barriers including fear of triggering spiritual distress, fear around answering questions about personal faith beliefs, and differences in personal beliefs between physicians and patients further exemplify neonatologists' lack of expertise or comfort in navigating these discussions.

Our study highlights a gap between the goal for prenatal counseling in cases of extreme prematurity and potentially life-limiting congenital anomalies and current practices that frequently exclude the values most important to many parents. To better support parents through the perinatal period, physicians must develop the skills to guide parents in articulating their values, including those of religion and spirituality, and then help to connect the medical information to what is most important to their family.^32–35^ Exploration and support of parental spirituality by social work, palliative care, and chaplaincy are necessary and important, yet may leave neonatologists unaware of the values that influence decision making.

For this reason, neonatologists should also possess the skills and responsibility to approach these topics in prenatal consultations and promote values-based shared decision making. Initial discussions by neonatologists may then lead to the involvement of palliative care and chaplaincy, whose expertise in this area can facilitate more in-depth exploration and longitudinal spiritual support. The findings of our study emphasize the need for more robust physician training in values exploration, including religion and spirituality, as well as a strengthened multidisciplinary approach to supporting values-based decision making for parents of high-risk infants.

## Limitations and Future Directions

There are several limitations to our study. Our study may be limited by our inclusion of spiritual terminology in documentation, which may exclude other aspects of spirituality including locus of control, connectedness, peace, meaning and purpose, and personal transformation. The most significant limitation to the study is a small sample size. Our findings may not accurately reflect the demographics, perspectives, practices, and perceived barriers of all neonatologists at our institution. Given that this was a single-institution study in a geographic region known for its religiosity, the results may not be generalizable to other locations or populations.

Future studies should include a larger more diverse population to better evaluate how differences in race, ethnicity, culture, and geography influence the exploration of parental RSF in prenatal consultations. Finally, our study only explores the physician perspective to prenatal consultations and does not address the parental perspective.

Future studies should seek to better understand the parental experience in prenatal counseling, including what information is remembered, what parents wish would have been discussed, what factors were most impactful, and whether they recognize if or how their personal values influenced decisions. Understanding the parental perspective may help guide future developments of the optimal approach to these conversations. It remains unknown how physicians and other medical team members may best approach discussions including RSF in the perinatal period.

Our research highlights the need to develop training programs to equip neonatologists and neonatology fellows with the skills to guide parents in identifying and applying their personal values to decision making. Future studies may also evaluate how a multidisciplinary team including physicians, spiritual care, palliative care, and social work can work together to explore parental values including spirituality, religion, and faith in the perinatal period.

## Conclusions

The prenatal consultation is often an emotionally laden experience for parents of high-risk infants. Although the goal of prenatal counseling is both to equip parents with information and partner in shared decision making, our study identified that these discussions frequently exclude what may be most important to many parents. Despite current practices and perceived barriers, our study also shows that neonatologists are interested in better understanding the role of parental values including RSF in the perinatal period and suggests a willingness to develop the skills to incorporate parental values into prenatal counseling for high-risk infants.

By recognizing the role and importance of RSF for many parents in the perinatal period and exploring this value in prenatal consultations of high-risk infants, neonatologists may enhance communication, better support parental decision making, and improve care for families in the NICU.

## Supplementary Material

Supplemental data

## Abbreviations Used

NICU: neonatal intensive care unit
RSF: religion, spirituality, and faith
