# Supplementary material for: Neonatologists' Perspectives on Exploring Parental Spirituality in Prenatal Consultations
Source: Palliat Med Rep. 2023 Mar 30;4(1):92–9. doi: 10.1089/pmr.2022.0052 (PMC10066782; doi:10.1089/pmr.2022.0052)
Supplement: Supplemental data [file Suppl_MaterialS1.zip › Manuscript Spiritual Terminology.docx]

KEY WORDS: SPIRITUAL TERMINOLOGY

- Religious affiliation: Baptist, Buddhist, Catholic, Christian, Islamic, The Church of Jesus Christ of Latter-Day Saints or “LDS”, Spiritual Non-Religious, Agnostic, Atheist
- Spiritual rites and practices: prayer, baptism, blessing, sacrament, crystals, attending religious services (mass, church, sacrament meeting), connecting to nature, practicing gratitude, focusing on beauty
- Support from faith leaders: bishop, priest, chaplain, elder
- Support from faith community: church congregation, LDS ward, LDS volunteers
- Maintaining hope and faith, belief in miracles, hoping for a miracle
- Surrendering into God’s hands, acceptance of God’s plan, belief in a “Greater Power” or “Higher Power”
