# Supplementary material for: Neonatologists' Perspectives on Exploring Parental Spirituality in Prenatal Consultations
Source: Palliat Med Rep. 2023 Mar 30;4(1):92–9. doi: 10.1089/pmr.2022.0052 (PMC10066782; doi:10.1089/pmr.2022.0052)
Supplement: Supplemental data [file Suppl_MaterialS1.zip › Manuscript_Survey.docx]

SURVEY

1. Demographics
   1. What is your age?
      1. 30 or younger
      2. 31-40
      3. 41-50
      4. 51-60
      5. 61 and older
   2. Please indicate your gender.
      1. Male
      2. Female
      3. Non-binary/third gender
      4. Prefer not to say
   3. What is your role in Neonatology?
      1. Attending
      2. Fellow
   4. How many years of practice have you had in your current role?
      1. 5 or less years
      2. 6-10 years
      3. 11-15 years
      4. 16 or more years
   5. Do you consider yourself spiritual and/or religious? Check all that apply.
      1. Spiritual
      2. Religious
      3. Neither
      4. (Optional) If so, do you identify with a specific religion or faith community? (fill in blank)
   6. How important is religion, spirituality, and/or faith in your own life?
      1. Very important
      2. Slightly important
      3. Neutral
      4. Unimportant
      5. Very unimportant
   7. In general, how important is religion, spirituality, and/or faith in your clinical practice?
      1. Very important
      2. Slightly important
      3. Neutral
      4. Unimportant
      5. Very unimportant
2. Attitudes and Perspectives
   1. In prenatal consultations for high-risk infants, how often do you explore parents’ religion, spirituality, and/or faith?
      1. Always
      2. Often
      3. Sometimes
      4. Rarely
      5. Never
   2. In your opinion, how important is it for neonatologists to be aware of parents’ values including religion, spirituality, and/or faith as it pertains to decision-making for the care of high-risk infants?
      1. Very important
      2. Slightly important
      3. Neutral
      4. Unimportant
      5. Very unimportant
   3. In your opinion, whose role is it to explore parents’ religion, spirituality, and faith during the perinatal period? (Check boxes)
      1. Neonatologist
      2. MFM
      3. Bedside nurse
      4. Social Work
      5. Spiritual care/chaplain
      6. Palliative care
      7. Other (fill in blank)
      8. Not important to explore
   4. In your opinion, what are the **three** most significant barriers to exploring religion, spirituality, and faith in prenatal consultations of high-risk infants?
      1. Lack of training or education in spiritual care
      2. Insufficient time
      3. Discomfort
      4. Differences in religion, spirituality, and faith between physicians and patients
      5. Fear of triggering spiritual distress in patients
      6. Fear of losing control of the conversation
      7. Fear around answering questions about personal faith beliefs
      8. It is not within the physician’s role
      9. Other (fill in the blank)
      10. No barriers perceived
      11. This is not necessary to explore in the prenatal consultation
   5. For you personally, what are the **three** most significant barriers to exploring religion, spirituality, and faith in prenatal consultations of high-risk infants?
      1. Lack of training or education in spiritual care
      2. Insufficient time
      3. Discomfort
      4. Differences in religion, spirituality, and faith between me and my patients
      5. Fear of triggering spiritual distress in patients
      6. Fear of losing control of the conversation
      7. Fear around answering questions about personal faith beliefs
      8. It is not within my role
      9. Other (fill in the blank)
      10. No barriers perceived
      11. This is not necessary to explore in the prenatal consultation
3. If offered, would you be interested in learning more about the role of values including religion, spirituality, and faith in parental decision making, coping, meaning making, etc.?
   1. Yes
   2. No
